# Association of demographic characteristics with evolution of interest in anesthesiology during medical school

**DOI:** 10.1371/journal.pone.0358122

**Published:** 2026-09-11

**Authors:** Max Y. Lu, Alexandra M. Hajduk, Jeph Herrin, Mytien Nguyen, Dowin Boatright, Sarwat I. Chaudhry

**Affiliations:** 1 Yale School of Medicine, New Haven, Connecticut, United States of America; 2 Section of Geriatrics, Department of Medicine, Yale School of Medicine, New Haven, Connecticut, United States of America; 3 Section of Cardiology, Department of Medicine, Yale School of Medicine, New Haven, Connecticut, United States of America; 4 Department of Immunobiology, Yale School of Medicine, New Haven, Connecticut, United States of America; 5 Department of Emergency Medicine, New York University Grossman School of Medicine, New York, New York, United States of America; 6 Section of General Internal Medicine, Department of Medicine, Yale School of Medicine, New Haven, Connecticut, United States of America; The Ohio State University College of Medicine, UNITED STATES OF AMERICA

## Abstract

**Background:**

Physician workforce diversity improves access to care, outcomes, and workforce productivity. Yet, women and sexual minorities remain underrepresented in anesthesiology, while representation for low-income trainees remains poorly characterized. How interest in anesthesiology evolves among these groups during medical school remains unclear.

**Methods:**

This retrospective cohort study of U.S. MD-granting medical school matriculants from 2014–2017 used Association of American Medical Colleges data on self-reported sociodemographic characteristics and anesthesiology interest. Students were grouped by interest evolution from matriculation to graduation: never interested (no-no), cultivated (no-yes), lost (yes-no), sustained (yes-yes). Chi-square tests characterized sociodemographic differences across interest paths. Firth logistic regression models assessed odds of cultivated vs never interested and sustained vs lost paths across sociodemographic groups.

**Results:**

Among 39,910 students in the cohort, 295 (0.7%) sustained, 2,007 (5.0%) cultivated, 494 (1.2%) lost interest, and 37,114 (93.0%) were never interested in anesthesiology. Women were less likely than men to have initial interest (1.5% vs 2.5%, *p* < .001), whereas initial interest among low-income (2.1% vs 1.9%, *p* = .087) and sexual minority (1.8% vs 2.0%, *p* = .505) students did not significantly differ from majority counterparts. Sustained interest did not differ significantly between women and men (OR: 0.85, 95% CI: 0.63–1.14), according to low-income (OR: 0.89, 95% CI: 0.65–1.22), or sexual minority status (OR: 0.77, 95% CI: 0.41–1.45). Women (OR: 0.50, 95% CI: 0.45–0.55) and sexual minority students (OR: 0.79, 95% CI: 0.65–0.96) were less likely to cultivate interest than men and heterosexual students, respectively. Low-income students had greater (OR: 1.27, 95% CI: 1.16–1.40) odds of cultivating interest compared to non-low-income peers.

**Conclusions:**

Women’s underrepresentation in anesthesiology is associated with lower initial and cultivated interest. Sexual minority students also demonstrated lower cultivated interest. These disparities identify time points in training where interest diverges by demographic group and may help prioritize where future research — including studies of targeted interventions — is most needed.

## Introduction

Diversity of the physician workforce is crucial to promote improved access to care, outcomes, and workforce productivity [1,2]. Furthermore, exposure to diverse colleagues can reduce implicit biases towards minority groups [3]. Diversity is also self-reinforcing, promoting further physician workforce diversification. Within anesthesiology, the presence of diverse faculty and mentors is often cited as an important influence on specialty choice [4]. Yet, women are less likely to apply into anesthesia and still comprise just 34% of faculty [5,6]. Additionally, compared with their representation within the overall medical student population, sexual minority students remain more poorly represented in anesthesia [7]. While low-income students remain underrepresented in surgery, data on their representation in anesthesia remains lacking [8]. Additionally, low-income students experience higher attrition rates during medical school and perceive lower personal and professional development support from their medical schools, highlighting challenges in professional development in this group [9,10].

Medical school is a critical period for the development of interest in anesthesiology: over 70% of medical students will change specialty interest in medical school [11,12]. It is therefore relevant to understand patterns of evolution in interest in anesthesiology during medical school. Prior work suggests women tend to avoid male-dominated specialties due to lack of initial and cultivated interest, though anesthesiology-specific patterns have not been studied [13,14]. Evolution of interest in anesthesiology during medical school has not been characterized in women, sexual minority, or low-income students, including cultivated interest in students who do not matriculate with interest and sustained interest in those who do matriculate with interest.

To better understand how sociodemographic backgrounds may relate to students’ interest and representation in anesthesiology, we evaluated the association of sociodemographic identities, including sex, income background, and sexual orientation, with evolution of interest in medical school from matriculation to graduation. We hypothesized women would have lower initial and cultivated (interest at graduation but not matriculation) interest but similar rates of loss of interest compared with men, while sexual orientation minority and low-income students would have lower rates of cultivated and sustained (interest at both matriculation and graduation) interest compared with heterosexual and non-low-income counterparts respectively.

## Methods

### Data and participants

We conducted a retrospective cohort study of U.S. MD-granting medical students who applied to medical school from the 2013–2014 through 2016–2017 application cycles and graduated by the 2023–2024 academic year. We utilized a data set containing deidentified individual student data (accessed June 23, 2025) obtained from the Association of American Medical Colleges data warehouse which contained data from the American Medical College Application Service (AMCAS), Matriculating Student Questionnaire (MSQ) [15], Year 2 Questionnaire (Y2Q) [16], and Graduation Questionnaire (GQ) [17]. The MSQ is administered in students’ first year of medical school while the GQ is administered from February to the beginning of June [15,17].

The study population included MD students who completed the MSQ during the 2014–2017 survey years and graduated from medical school by 2024. Students who did not provide a response to the specialty interest item on the MSQ or the intended specialty item on the GQ were excluded. Responses indicating an undecided specialty or no intention to practice medicine were considered valid responses and were retained.

Our analyses were conducted according to the Strengthening the Reporting of Observational Studies in Epidemiology (STROBE) reporting guideline [18]. This study was deemed exempt as not human subjects research by the Yale University Institutional Review Board.

### Sociodemographic characteristics

Students self-reported sex on their AMCAS application [19]. Sexual orientation was based on students’ responses on the MSQ in which they specified if they identified as heterosexual, bisexual, gay or lesbian. Childhood socioeconomic disadvantage was defined based on three variables on the AMCAS application, with students being classified as low-income if they met any one of the following criteria: 1) reported receipt of a Pell grant; 2) receipt of state or federal assistance (i.e., Supplemental Nutritional Assistance Program); or 3) self-reported childhood household income less than $50,000 per year (approximately corresponding to the bottom two quintiles of U.S. household income) and in alignment with prior literature [20,21].

### Endpoints

The endpoint, interest in anesthesiology, was defined based on questions in the MSQ and GQ in which students were asked to select which specialty they were most likely to pursue or which they intended to pursue, respectively. We defined “sustained interest” as interest in anesthesiology on both the MSQ and GQ. “Cultivated interest” was defined as no interest on the MSQ but expression of interest on the GQ. “Lost interest” was defined as expression of interest on the MSQ but not the GQ and “never interested” was defined as no expression of interest on both the MSQ and the GQ.

### Statistical analysis

We summarized the characteristics of the cohort, including rates of missingness, by change in interest in anesthesiology category, testing for differences across categories using chi-square tests of independence in sex, income background, and sexual orientation groups. We also used one-sample proportion tests to compare the composition of students interested in anesthesiology at graduation to the overall medical school population.

To account for potential nonresponse bias due to missing data, we employed multiple imputations by chained equations in R utilizing the assumption of Missing at Random. Variables imputed included: childhood family income, MSQ sexual orientation, Pell Grant receipt, state/federal financial assistance receipt, and sex. Variables were imputed using logistic regression for binary variables and polytomous regression for multi-category variables. We also included sexual orientation on the GQ and Y2Q, degree program type, MSQ anesthesiology interest, GQ anesthesiology interest, Y2Q anesthesiology interest, and ethnicity to improve imputation accuracy. A total of 55 imputed datasets were generated.

Within each imputed dataset, composite variables were re-derived for income background, and sexual minority status. Following von Hippel (2007) [22], we then employed a Multiple Imputation then Deletion (MID) approach wherein imputed endpoint values were used to improve imputation accuracy of missing covariates but subsequently dropped. Cases originally missing GQ or MSQ interest data were therefore included in the imputation models but excluded prior to fitting the outcome regressions. This avoided imputation of outcomes while still using information from those cases in the imputation models. Results from each imputation were combined using Rubin’s Rules to obtain overall estimates and 95% confidence intervals [23].

Although events-per-variable were >10 for all analyses, unadjusted Firth penalized logistic regression was employed due to small cell sizes and concerns regarding model instability and non-convergence to examine associations between sociodemographic factors (sex, socioeconomic status, and sexual orientation) and paths of interest in anesthesiology: (1) sustained versus lost interest among those initially interested and (2) cultivated versus never interested among those without initial interest. For each model, the reference group was students with lost interest or who were never interested, respectively. Calculation of variance inflation factors on the corresponding standard logistic regression models indicated minimal collinearity. Due to small subgroup sizes, students were grouped as sexual minority (gay, lesbian, or bisexual) vs heterosexual for regression.

For each model, we estimated marginal predicted probabilities for each predictor using a simulation-based marginal standardization approach [24,25]. Specifically, for each model, we drew 2,000 samples from the distribution of the estimated coefficients. For a given predictor level, we fixed the predictor for all observations at that level while holding other predictors at their observed values, calculating the average predicted probability across the cohort for each simulation draw. Marginal predicted probabilities were also pooled across imputations using Rubin’s Rules [23].

All tests were two-tailed. *P*-values < .05 were considered statistically significant. Statistical analysis was performed using R statistical software version 4.2.2.

### Sensitivity analysis

To assess potential within-school correlation of outcomes, we applied a cluster bootstrap procedure to a single imputed dataset. Schools were resampled with replacement (2,000 resamples), and Firth penalized logistic regression models were refit within each resample, with MID applied within each iteration to preserve the original missingness pattern of the MSQ and GQ specialty outcomes.

## Results

### Cohort derivation

**Fig 1:**
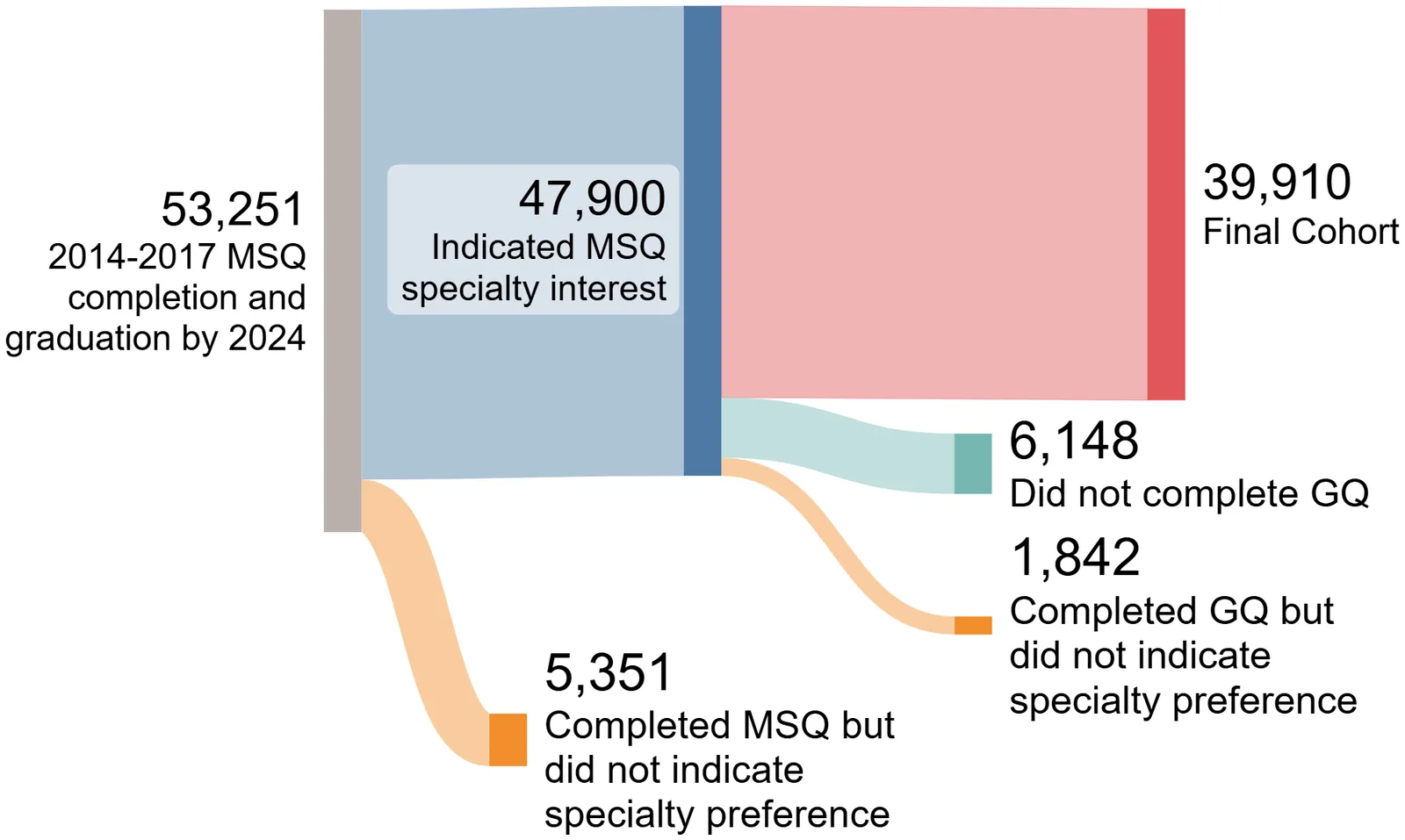
Sankey diagram for inclusion in cohort. Sankey diagram showing sequential exclusion of U.S. medical students completing the 2014-2017 MSQ who graduated by 2024. Out of 53,251 students, exclusions for missing MSQ specialty interest, GQ noncompletion, or missing GQ specialty intention resulted in a final cohort of 39,910 students.

There were 53,251 MD students who completed the 2014–2017 MSQ and graduated by 2024 (Fig 1). Of those students, 5,351 (10.0%) were excluded for not indicating specialty interest on the MSQ. A further 7,990 (15.0%) were excluded for lack of data on intended specialty on the GQ, of which 6,148 (11.5%) did not complete the GQ, and 1,842 (3.5%) completed the GQ but did not indicate specialty intention, leaving a final study cohort of 39,910 (74.9%). Included and excluded students differed with respect to sex, sexual orientation, and socioeconomic background (S1 Table).

### Cohort characteristics

Among the students in the study cohort, 21,095 (52.9%) were female (Table 1). Additionally, 12,687 (31.8%) of the cohort were from a low-income background (distribution of socioeconomic status variables in S2 Table). 1,347 (3.4%) were gay or lesbian, 1,185 (3.0%) were bisexual, and 36,901 (92.5%) were heterosexual or straight. Of the cohort 117 (0.3%) graduated in 3 years, 33,860 (84.8%) graduated in 4 years, 4,750 (11.9%) in 5 years, 543 (1.4%) in 6 years, 210 (0.5%) in 7 years, 341 (0.9%) in 8 years, and 89 (0.2%) in 9 years.

**Table 1:** Characteristics of study cohort by interest in anesthesiologyanesthesiology.

Demographic Characteristic	All students (N=39,910)	Never Interested (N=37,114)	Cultivated Interest (N=2,007)	Sustained Interest (N=295)	Lost Interest (N=494)	*P*
Sex						<.001
Male	18,806 (47.1%)	17,072 (46.0%)	1,267 (63.1%)	181 (61.4%)	286 (57.9%)	
Female	21,095 (52.9%)	20,033 (54.0%)	740 (36.9%)	114 (38.6%)	208 (42.1%)	
Declined to Respond/Unknown	9 (<0.1%)	9 (<0.1%)	0 (0.0%)	0 (0.0%)	0 (0.0%)	
Low-income						<.001
Yes	12,687 (31.8%)	11,669 (31.4%)	746 (37.2%)	98 (33.2%)	174 (35.2%)	
No	26,619 (66.7%)	24,887 (67.1%)	1,231 (61.3%)	195 (66.1%)	306 (61.9%)	
Missing Data	604 (1.5%)	558 (1.5%)	30 (1.5%)	2 (0.7%)	14 (2.8%)	
Sexual Orientation						.005
Heterosexual or straight	36,901 (92.5%)	34,294 (92.4%)	1,873 (93.3%)	279 (94.6%)	455 (92.1%)	
Bisexual	1,185 (3.0%)	1,137 (3.1%)	34 (1.7%)	5 (1.7%)	9 (1.8%)	
Gay or lesbian	1,347 (3.4%)	1,239 (3.3%)	77 (3.8%)	10 (3.4%)	21 (4.3%)	
Unknown	477 (1.2%)	444 (1.2%)	23 (1.1%)	1 (0.3%)	9 (1.8%)	
Sexual Minority						.340
Yes	2,532 (6.3%)	2,376 (6.4%)	111 (5.5%)	15 (5.1%)	30 (6.1%)	
No	36,901 (92.5%)	34,294 (92.4%)	1,873 (93.3%)	279 (94.6%)	455 (92.1%)	
Unknown	477 (1.2%)	444 (1.2%)	23 (1.1%)	1 (0.3%)	9 (1.8%)	

Percentages are displayed as column percentages and may not sum to 100% due to rounding. Unknown sexual orientation reflects nonresponse rather than explicit “prefer not to respond.”

### Evolution of anesthesiology interest

Of the cohort, 789 (2.0%) initially reported anesthesia as their most likely specialty choice at matriculation (sustained and lost interest groups) while 39,121 (98.0%) did not (Table 1). At graduation, 2,302 (5.8%) students intended to enter anesthesiology (cultivated and sustained interest groups), while 37,608 (94.2%) did not. Far more students had cultivated (N = 2,007; 5.0%) than sustained (N = 295; 0.7%) or lost (N = 494; 1.2%) interest (Table 1).

### Anesthesiology pathways by sex

Female students were less likely than male counterparts to have initial interest (1.5% vs 2.5%, *p* < .001) and were underrepresented among students expressing interest in anesthesiology at graduation compared with their proportion in the overall medical student body (37.1% vs 52.9%, *p* < .001). Among those without initial interest, female students had lower odds of cultivating interest compared to male counterparts (Table 2). Among those with initial anesthesiology interest, female students did not have significantly lower odds of sustaining interest. Translating these associations into predicted probabilities, among those without initial anesthesiology interest, female students had a 3.6% chance to cultivate interest (compared to 6.9% among male students; S3 Table). Female students with initial anesthesiology interest had a 35.3% chance of sustained interest compared to 39.1% among male students.

**Table 2:** Association of selected demographic characteristics with career path in anesthesiology.

Demographic Characteristic	Cultivated Interest vs Never-Interested (ref) OR (95% CI)	*P*	Sustained vs Lost (ref) OR (95% CI)	*P*
Sex				
Female (ref: male)	0.50 (0.45-0.55)	< .001	0.85 (0.63-1.14)	.286
Low-income				
Yes (ref: non-low-income)	1.27 (1.16-1.40)	< .001	0.89 (0.65-1.22)	.467
Sexual Orientation				
Sexual Minority (Bisexual, gay, or lesbian) (ref: heterosexual or straight)	0.79 (0.65-0.96)	.017	0.77 (0.41-1.45)	.419

Results of penalized Firth regression models with interest path as dependent variable.

### Anesthesiology pathways by income background

Low-income (2.1% vs 1.9%, *p* = .087) students were not less likely to have initial interest compared to non-low-income counterparts and were better represented among students expressing interest in anesthesiology at graduation compared to their overall medical school representation (37.2% vs 32.3%, *p* < .001). Among those without initial anesthesiology interest, low-income students had a 5.9% chance to cultivate interest in anesthesiology (S3 Table), compared to 4.7% among non-low-income students, demonstrating greater odds of cultivated interest than non-low-income counterparts (Table 2). Among those with initial anesthesiology interest, low-income students had a 35.9% predicted probability to sustain interest, compared to 38.5% among non-low-income students, demonstrating no significant difference in odds of sustaining interest.

### Anesthesiology pathways by sexual orientation

Sexual minority (1.8% vs 2.0%, *p* = .505) students were not less likely to have initial interest compared to heterosexual peers. At graduation, their proportion amongst those interested in anesthesiology also did not significantly differ (5.5% vs 6.4%, *p* = .091) from their representation in the overall medical student population. Among those without initial interest, a 4.2% chance of sexual minority students to cultivate interest, compared with 5.2% among non-sexual minority students reflects lower odds for sexual minority students to cultivate interest (Table 2 and S3 Table). Among those with initial interest, sexual minority students had a 32.4% predicted probability of sustained interest compared to 37.9% for heterosexual students, reflecting that odds of sustained interest did not significantly differ (Table 2).

Across demographic groups, accounting for potential clustering by matriculation school via cluster bootstrap resampling yielded estimates similar to the primary analysis with respect to confidence interval width and point estimates (S4 Table).

## Discussion

In this study, women had lower initial interest in anesthesiology, lower odds of cultivating interest in anesthesiology during medical school, and similar odds of sustaining interest compared to male students. Low-income students did not differ in initial interest, had greater odds of cultivating interest compared to non-low-income counterparts, without significant differences in odds of sustained interest. Sexual minority students did not differ in initial interest or odds of sustained interest but had lower odds of cultivated interest but compared to heterosexual peers. Lastly, in our cohort, just 295/2,302 (12.8%) of students interested in anesthesiology at graduation had expressed interest at matriculation (i.e., sustained interest), lower than the roughly 26% each year nationally across all specialties [26], suggesting far greater cultivation of interest in anesthesiology compared with other specialties.

These findings build on prior work which demonstrates that women have lower interest in anesthesiology [5], and that women tend not to enter male-dominated specialties due to lower initial and cultivated interest, not greater loss of interest [13,14]. Similarly, higher cultivated interest without lower sustained interest among low-income students aligns with prior findings showing students with history of housing and food insecurity are more likely to apply to non-surgical specialties such as anesthesiology [27].

Reduced interest cultivation among female students may reflect, in part, mentorship and structural factors. Female mentors are often influential in specialty choice [4,28], yet comprise just 34% of faculty and are further underrepresented in leadership roles and higher academic ranks [6,29–31]. Female anesthesiologists often report career impacts on family planning [32,33], inflexible policies and negative attitudes relating to child-rearing [32–34], and the highest rate of maternal discrimination amongst specialties in one study (47.1%) [35]. Anesthesiology, despite its reputation as a lifestyle specialty, involves early mornings, overnight calls, and long shifts. Women in anesthesiology are at higher risk of burnout and depression than male counterparts [36,37], which in addition to motherhood-related challenges, may also be related to greater gender-based discrimination or sexual harassment [4,32,37,38]. A persistent gender-based pay gap also persists [39], and in one survey of 502 female anesthesiologists, 52% felt gender negatively affected career advancement, and 90% felt they needed to work harder than men to achieve similar goals [32].

Lower odds of cultivating interest for sexual minority students may reflect sexual orientation-related discrimination. One survey of anesthesiology trainees found that 4.4% experienced sexual orientation-related discrimination and 3.7% reported their sexual orientation negatively impacted pursuit of anesthesiology [4]. LGBTQ+ faculty are more likely to occupy lower academic ranks [30], and are at greater risk for burnout [40]. Furthermore, one study of 8 specialties found that anesthesiology was rated the third least accepting of sexual minorities amongst medical students and was just one of two specialties where sexual minority students felt significantly less comfortable in applying compared to non-sexual minority counterparts [41]. Trainee perceptions may also be influenced by the broader operating room climate. Surgical subspecialties are consistently perceived as the least inclusive towards sexual minorities [41,42], which may be compounded by operating room support staff attitudes [43].

Greater odds of cultivated interest among low-income students may reflect the lower away rotation costs compared to other specialties [44]. Though insufficient to diminish anesthesiology interest, low-income students still experience barriers such as higher attrition, perceive lower school support of personal and professional development [9,10], and remain underrepresented in anesthesia and medical school overall [20].

Building on the patterns observed here and in prior literature, we highlight several opportunities that merit further study to foster equity in anesthesiology recruitment, starting with medical school curricula. While clerkship experiences strongly influence in specialty choice [45,46], just 16% of medical schools report required anesthesiology rotations [47]. Despite the influence of mentorship in specialty choice [45,48], 69% of anesthesiology clerkship programs reported that they do not train teachers at all and 38% reported no faculty renumeration for teaching [47]. Greater support for medical student teaching and exposure to anesthesiology in curriculums may promote interest in groups with lower initial and cultivated interest such as women and sexual minorities. Additionally, institutional responses such as salary transparency, standardized leave policies, protected lactation spaces, community-building events, and strong anti-harassment enforcement may be key to improving recruitment and retention for female faculty trainee mentors [49–51]. For low-income students, the advent of program signaling which has decreased application volume and continued absence of financial barriers such as mandatory away rotations and in-person interviews may help reduce obstacles [52,53].

As the field of anesthesiology increases in competitiveness, potential new barriers warrant monitoring. Women have fewer publications even after adjusting for academic metrics, raising concerns about potential disparities as research expectations grow [54,55]. Although applicants with histories of housing or food insecurity do not have lower research output [27], the Pass/Fail STEP 1 transition and increasing specialty competitiveness warrants monitoring of whether emphasis will shift towards financially burdensome away rotations [56], though increased funding to support students may mitigate such disparities [57].

Given the descriptive nature of our analysis, we did not adjust for interest evolution predictors such as race/ethnicity or intersectionality [5,8], nor did we apply formal correction for multiple comparisons across our chi-square and proportion tests, as these were used to characterize the cohort rather than to test inferential hypotheses. Likewise, we did not correct for multiple comparisons for our two regression models which were pre-specified rather than derived via exploratory analyses and address conceptually distinct questions.

Our study does have several limitations. The group initially interested in anesthesia (sustained vs lost) was far smaller (N = 789) than the group initially not interested (cultivated vs never interested) (N = 39,121), contributing to wider confidence intervals and less precise estimates despite use of Firth penalized regression. Furthermore, the survey instruments assessed sex as a binary variable rather than gender and provided limited options for sexual orientation, and we created the umbrella category of “sexual minority” to increase regression power and stability, restricting our ability to capture the full effects of gender identity and sexual orientation diversity.

Interest pathway also relied on responses to the MSQ, in which students identify a single ‘most likely’ specialty at matriculation. These early preferences may reflect limited clinical exposure or pre-medical influences, rather than well-informed career interests. Our graduation interest endpoint also does not equal specialty entry, as though across specialties low-income students match at similar rates [21], within anesthesiology women match at higher rates than men [5].

Despite the use of MID, our results are still subject to selection and nonresponse bias. Students who did not complete the MSQ or GQ or did not indicate a specialty were not included, which may preferentially omit individuals who are less engaged or more uncertain in their career preferences. In our cohort, male and low-income students were more likely to be excluded (S1 Table), and while the pathways of interest these students may have followed are unknown, our results should be interpreted with caution. Students who were excluded for failure to indicate specialty intention on the GQ also had higher rates of missing sexual orientation data, potentially reflecting broader patterns of survey nonresponse, as reductions were observed proportionally across all reported sexual orientation categories.

The generalizability of our findings is further limited by cohort selection. Our sample including 2014–2017 matriculants who graduated by 2024 may underrepresent MD/PhD students in later entry years, given the protracted training time of these students. Our dataset also did not include osteopathic medical students. While there exists little literature on anesthesiology interest development in this group, they are less likely to have anesthesiology programs affiliated with their medical school, which may be detrimental to women and sexual minorities who have lower initial and cultivated interest in anesthesiology and value diverse mentors in fostering specialty interest [4,56].

Future qualitative or mixed-methods studies are needed to better understand the experiences underlying differences in interest development. Updated datasets with more inclusive measures of gender and sexual identity, more entry years, and osteopathic medical students may enhance power to detect outcomes as well as generalizability. Further research should also examine how evolving aspects of the residency application process—such as program signaling, virtual interviews, and increasing competitiveness—affect accessibility.

This study highlights significant disparities in how interest in anesthesiology develops and is sustained across medical school, particularly among women and sexual minority students. By identifying key stages where interest is most likely to be lost or gained, these results may help prioritize where future causal and interventional research is needed to foster a more diverse and representative anesthesiology workforce.

## Supporting information

S1 Table Sociodemographic characteristics of included vs excluded participants. Comparison of characteristics of included (N = 39,910) vs excluded (N = 13,341) participants among students who completed the 2014–2017 MSQ and graduated by 2024 with calculation of standardized mean differences (SMD). A |SMD| > 0.10 is considered indicative of meaningful imbalance between groups. Excluded students comprised individuals who did not report intended specialty on the Graduation Questionnaire (GQ). Percentages may not sum to 100% due to rounding.
(DOCX)

S2 Table Socioeconomic status variable distribution of cohort. The 604 students with missing data in all three variables are not included in this table. Percentages may not sum to 100% due to rounding.
(DOCX)

S3 Table Marginal predicted probabilities for anesthesiology career paths. Average predicted probabilities of cultivated interest among those without initial interest in anesthesiology and sustained interest among those initially interested in anesthesiology. P-values are based on the corresponding Firth regressions.
(DOCX)

S4 Table Cluster Bootstrap Sensitivity Analysis for School-Level Clustering. Sensitivity analysis assessing the influence of school-level clustering on Firth logistic regression estimates for the association of sex, income background, and sexual orientation with anesthesiology interest pathways. A cluster bootstrap (2,000 resamples of schools with replacement) was performed on a single completed dataset from the primary multiple imputation.
(DOCX)
