# Supplementary material for: Association of demographic characteristics with evolution of interest in anesthesiology during medical school
Source: PLoS One. 2026 Sep 11;21(9):e0358122. doi: 10.1371/journal.pone.0358122 (PMC13567819; doi:10.1371/journal.pone.0358122)
Supplement: Supplementary file 1 [file pone.0358122.s001.docx]

**S1 Table.** **Sociodemographic Characteristics of Included vs Excluded Participants**

| Sociodemographic Characteristic | Included Students  N=39,910  (% of included students) | Excluded Students  N=13,341  (% of excluded students) | Standardized Mean Difference |
| --- | --- | --- | --- |
| Sex |  |  |  |
| Male | 18,806 (47.1%) | 7,096 (53.2%) | **-0.122** |
| Female | 21,095 (52.9%) | 6,240 (46.8%) | **0.122** |
| Declined to Respond/Unknown | 9 (<0.1%) | 5 (<0.1%) | -0.009 |
| Sexual Orientation |  |  |  |
| Heterosexual or straight | 36,901 (92.5%) | 7,461 (55.9%) | **0.919** |
| Bisexual | 1,185 (3.0%) | 240 (1.8%) | 0.077 |
| Gay or lesbian | 1,347 (3.4%) | 223 (1.7%) | 0.109 |
| Missing | 477 (1.2%) | 5,417 (40.6%) | **-1.108** |
| Pell Grant Receipt |  |  |  |
| Yes | 8,469 (21.2%) | 3,439 (25.8%) | **-0.108** |
| No | 29,932 (75.0%) | 9,158 (68.6%) | **0.142** |
| Missing | 1,509 (3.8%) | 744 (5.6%) | -0.085 |
| State/Federal Assistance Receipt |  |  |  |
| Yes | 7,581 (19.0%) | 3,033 (22.7%) | -0.092 |
| No | 28,388 (71.1%) | 8,706 (65.3%) | **0.126** |
| Unknown/Decline to Answer | 3,337 (8.4%) | 1,259 (9.4%) | -0.038 |
| Missing | 604 (1.5%) | 343 (2.6%) | -0.075 |
| Income |  |  |  |
| <50k/yr | 6,239 (15.6%) | 2,675 (20.1%) | **-0.116** |
| ≥50k/yr | 26,065 (65.3%) | 7,821 (58.6%) | **0.138** |
| Unknown/Decline to Answer | 7,002 (17.5%) | 2,502(18.8%) | -0.031 |
| Missing | 604 (1.5%) | 343 (2.6%) | -0.075 |

Comparison of characteristics of included (N=39,910) vs excluded (N=13,341) participants among students who completed the 2014-2017 MSQ and graduated by 2024 with calculation of standardized mean differences (SMD). A |SMD| > 0.10 is considered indicative of meaningful imbalance between groups. Excluded students comprised individuals who did not report intended specialty on the Graduation Questionnaire (GQ). Percentages may not sum to 100% due to rounding.
