# Supplementary material for: Association of demographic characteristics with evolution of interest in anesthesiology during medical school
Source: PLoS One. 2026 Sep 11;21(9):e0358122. doi: 10.1371/journal.pone.0358122 (PMC13567819; doi:10.1371/journal.pone.0358122)
Supplement: Supplementary file 2 [file pone.0358122.s002.docx]

**S2 Table. Socioeconomic Status Variable Distribution of Cohort**

| Demographic Characteristic | Low-income (N=12,687) | Non-low-income (N=26,619) |
| --- | --- | --- |
| Pell Grant Receipt |  |  |
| Yes | 8,469 (66.8%) | 0 (0.0%) |
| No | 3,938 (31.0%) | 25,994 (97.7%) |
| Missing | 280 (2.2%) | 625 (2.3%) |
| Childhood Household Income |  |  |
| <50k | 6,239 (49.2%) | 0 (0.0%) |
| ≥50k | 5,274 (41.6%) | 20,791 (78.1%) |
| Decline to Answer | 205 (1.6%) | 2,171 (8.2%) |
| Unknown | 969 (7.6%) | 3,657 (13.7%) |
| State/Federal Assistance Receipt |  |  |
| Yes | 7,581 (59.8%) | 0 (0.0%) |
| No | 4,085 (32.2%) | 24,303 (91.3%) |
| Decline to Answer | 76 (0.6%) | 499 (1.9%) |
| Unknown | 945 (7.4%) | 1,817 (6.8%) |

The 604 students with missing data in all three variables are not included in this table. Percentages may not sum to 100% due to rounding.
