# Supplementary material for: Association of demographic characteristics with evolution of interest in anesthesiology during medical school
Source: PLoS One. 2026 Sep 11;21(9):e0358122. doi: 10.1371/journal.pone.0358122 (PMC13567819; doi:10.1371/journal.pone.0358122)
Supplement: Supplementary file 3 [file pone.0358122.s003.docx]

**S3 Table. Marginal Predicted Probabilities for Anesthesiology Career Paths**

| Demographic Characteristic | Cultivated Interest % (95%) | P-value | Sustained Interest % (95% CI) | P-value |
| --- | --- | --- | --- | --- |
| Sex |  | < .001 |  | .286 |
| Male | 6.9% (6.5-7.3%) |  | 39.1% (34.6-43.5%) |  |
| Female | 3.6% (3.3-3.8%) |  | 35.3% (30.1-40.5%) |  |
| Low-income |  | < .001 |  | .467 |
| No | 4.7% (4.5-5.0%) |  | 38.5% (34.2-42.8%) |  |
| Yes | 5.9% (5.5-6.3%) |  | 35.9% (30.3-41.4%) |  |
| Sexual Orientation |  | .017 |  | .419 |
| Heterosexual | 5.2% (5.0-5.4%) |  | 37.9% (34.4-41.3%) |  |
| Sexual minority (Bisexual, gay or lesbian) | 4.2% (3.4-4.9%) |  | 32.4% (19.3-45.4%) |  |

Average predicted probabilities of cultivated interest among those without initial interest in anesthesiology and sustained interest among those initially interested in anesthesiology. P-values are based on the corresponding Firth regressions.
