# Supplementary material for: Association of demographic characteristics with evolution of interest in anesthesiology during medical school
Source: PLoS One. 2026 Sep 11;21(9):e0358122. doi: 10.1371/journal.pone.0358122 (PMC13567819; doi:10.1371/journal.pone.0358122)
Supplement: Supplementary file 4 [file pone.0358122.s004.docx]

**S4 Table. Cluster Bootstrap Sensitivity Analysis for School-Level Clustering**

| Demographic Characteristic | Cultivated Interest vs Never-Interested (ref)  OR (95% CI) | *P* | Sustained vs Lost (ref)  OR (95% CI) | *P* |
| --- | --- | --- | --- | --- |
| Sex |  |  |  |  |
| Female (ref: male) | 0.50 (0.45-0.55) | <.001 | 0.84 (0.63-1.14) | .260 |
| Low-income |  |  |  |  |
| Yes (ref: non-low-income) | 1.25 (1.13-1.38) | <.001 | 0.87 (0.66-1.12) | .298 |
| Sexual Orientation |  |  |  |  |
| Sexual Minority (Bisexual, gay, or lesbian) (ref: heterosexual or straight) | 0.78 (0.62-0.96) | .015 | 0.74 (0.38-1.40) | .356 |

Sensitivity analysis assessing the influence of school-level clustering on Firth logistic regression estimates for the association of sex, income background, and sexual orientation with anesthesiology interest pathways. A cluster bootstrap (2,000 resamples of schools with replacement) was performed on a single completed dataset from the primary multiple imputation.
